# Comparison of cisplatin sensitivity and the 18F fluoro-2-deoxy 2 glucose uptake with proliferation parameters and gene expression in squamous cell carcinoma cell lines of the head and neck

**DOI:** 10.1186/1756-9966-28-17

**Published:** 2009-02-13

**Authors:** Eva Henriksson, Elisabeth Kjellén, Bo Baldetorp, Pär-Ola Bendahl, Åke Borg, Eva Brun, Fredrik Mertens, Tomas Ohlsson, Karin Rennstam, Johan Wennerberg, Peter Wahlberg

**Affiliations:** 1 Dept of Otorhinolaryngology, University Hospital Malmö, SE-205 02 Malmö, Sweden; 2 Dept of Oncology, Lund University Hospital, SE-221 85 Lund, Sweden; 3 Dept of Clinical Genetics, Lund University Hospital, SE-221 85 Lund, Sweden; 4 Dept of Radiation Physics, Lund University Hospital, SE-221 85 Lund, Sweden; 5 Dept of Otorhinolaryngology, Lund University Hospital, SE-221 85 Lund, Sweden

## Abstract

**Background:**

The survival of patients with locally advanced head and neck cancer is still poor, with 5-year survival rates of 24–35%. The identification of prognostic and predictive markers at the molecular and cellular level could make it possible to find new therapeutic targets and provide "taylor made" treatments. Established cell lines of human squamous cell carcinoma (HNSCC) are valuable models for identifying such markers.

The aim of this study was to establish and characterize a series of cell lines and to compare the cisplatin sensitivity and 18F fluoro-2 deoxy 2 glucose (18F-FDG) uptake of these cell lines with other cellular characteristics, such as proliferation parameters and TP53 and CCND1 status.

**Methods:**

Explant cultures of fresh tumour tissue were cultivated, and six new permanent cell lines were established from 18 HNSCC cases. Successfully grown cell lines were analysed regarding clinical parameters, histological grade, karyotype, DNA ploidy, and index and S-phase fraction (Spf). The cell lines were further characterized with regard to their uptake of 18F-FDG, their sensitivity to cisplatin, as measured by a viability test (crystal violet), and their TP53 and CCND1 status, by fluorescence in situ hybridization (FISH), polymerase chain reaction single-strand conformation polymorphism (PCR-SSCP) with DNA sequencing and, for cyclin D1, by immunohistochemistry.

**Results:**

Patients with tumours that could be cultured in vitro had shorter disease-free periods and overall survival time than those whose tumours did not grow in vitro, when analysed with the Kaplan-Meier method and the log-rank test. Their tumours also showed more complex karyotypes than tumours from which cell lines could not be established. No correlation was found between TP53 or CCND1 status and 18F-FDG uptake or cisplatin sensitivity. However, there was an inverse correlation between tumour cell doubling time and 18F-FDG uptake.

**Conclusion:**

In vitro growth of HNSCC cells seem to be an independent prognostic factor, with cell lines being more readily established from aggressive tumours, a phenomenon more dependent on the molecular genetic characteristics of the tumour cells than on tumour location or TNM status.

## Background

In 2006, 101,600 new cases and 42,400 deaths resulting from oropharyngeal cancer were registered in Europe [1]. Although morbidity has decreased, the outcome of patients with locally advanced head and neck cancer is still poor, 5-year survival rates being only 24–35% [2,3]. There is a need for more individualized, "taylor-made" therapies in order to avoid under-treatment (residual disease) as well as over-treatment (unnecessary morbidity). The application of new techniques has improved our understanding of the mechanisms behind the origin, maintenance and progression of tumours, and new insights have facilitated the identification of diagnostic, prognostic and predictive markers at molecular and cellular levels, paving the way for novel therapeutic approaches.

Cell lines of human squamous cell carcinoma are valuable models for identifying such markers, and for studies of tumour biology. In this study, explant cultures of fresh tumour tissue were cultivated and six new permanent cell lines were established from 18 patients with head and neck squamous cell carcinoma (HNSCC). The cell lines established in this study were used to test for cisplatin sensitivity, 18F-FDG uptake, as a measure of metabolic activity, and various other tumour characteristics.

## Methods

### Patients

Fresh tumour samples were collected during 1995–1999 from 18 patients with HNSCC. The patients participated voluntary and with informed consent. Seventeen of the 18 patients with HNSCC were previously untreated and one patient had a residual tumour after radiotherapy. Eight tumours were located in the oral cavity, four in the larynx, two in the nasopharynx, and one each in the oropharynx, hypopharynx and in the maxillary sinus. One was an untreated lymph node metastasis of unknown primary origin. Table 1 shows the tumour TNM (Tumour, Node, Metastasis) classification, stage, grade, ploidity and karyotype of each tumour. Permanent cell lines were successfully established from the first six tumours in Table 1; four were from the oral cavity, one from the maxillary sinus and one was a residual tumour from the oral cavity. Table 2 shows clinical information regarding treatment regim, survival data and causes of death.

**Table 1:** The characteristics of the primary tumours regarding clinical data, DNA content and cytogenetics.

**Tumour**	**Take Rate**	**Corresponding cell line name**	**Site**	**T_ stage**	**N_ stage**	**M_ stage**	**Stage**	**Grade**	**Flowcyto Metry**	**DNA _indices**	**Cytogenetics**
1	yes	LU-HNxSCC-3	310	4	0	0	4	G3	diploid	1	not complex
2	yes	LU-HNxSCC-6	021	3	0	0	3	G3	nondiploid	1,25	complex
3	yes	LU-HNxSCC-8	060	2	1	0	3	G3	nondiploid	1,9	complex
4	yes	LU-HNxSCC-4	040	2	0	0	2	G3	nondiploid	1,85	complex
5	yes	LU-HNxSCC-5	062	2	2b	0	4	G2	nondiploid	2,38	complex
6	yes	LU-HNxSCC-7	060	2	0	0	2	G2	diploid	1	complex
7	no		021	2	0	0	2	G2	nondiploid	1,9	failure
8	no		119	1	0	0	1	G3	nondiploid	1,22	failure
9	no		321	3	1	0	3	G3	diploid	1	not complex
10	no		040	2	2c	0	4	G2	nondiploid	1,87	complex
11	no		090	3	0	0	3	Gx	diploid	1	failure
12	no		322	4	0	0	4	G2	nondiploid	1,93	failure
13	no		119	2	2a	0	4	G4	diploid	1	failure
14	no		139	2	2c	0	4	G2	nondiploid	1,28	missing
15	no		321	4	2b	0	4	G3	nondiploid	1,59	complex
16	no		320	4	0	0	4	G2	diploid	1	failure
17	no		770	0	2b	0	4	G2	nondiploid	1,51	failure
18	no		040	1	2a	0	4	G2	diploid	1	complex

**Table 2:** The features of the primary tumours regarding treatment regime, follow up time and cause of death.

**Tumour**	**Take Rate**	**Corresponding cell line**	**Site**	**Surgery**	**Radiation-therapy**	**Disease free months**	**Overall survival In months**	**Death caused by intercurrent disease**	**Death caused by HNSCC**
1	yes	LU-HNxSCC-3	310	No	Yes	0	12	No	Yes

2	yes	LU-HNxSCC-6	021	Yes	Yes	6	8	no	Yes

3	yes	LU-HNxSCC-8	060	Yes	Yes	2	4	Yes	No

4	yes	LU-HNxSCC-4	040	Yes	Yes	37	42	yes	No

5	yes	LU-HNxSCC-5	062	No	Yes	4	4	No	Yes

6	yes	LU-HNxSCC-7	060	Yes	yes	19	25	no	Yes

7	no		021	Yes	No	0	1	Yes	No

8	no		119	No	Yes	25	43	No	Yes

9	no		321	No	No	0	1	No	Yes

10	no		040	Yes	Yes	74	96	No	Yes

11	no		090	No	Yes	99	108	No	No

12	no		322	Yes	Yes	85	87	Yes	No

13	no		119	No	Yes	90	108	No	No

14	no		139	No	Yes	0	78	No	Yes

15	no		321	Yes	Yes	66	75	No	Yes

16	no		320	Yes	Yes	8	1	Yes	No

17	no		770	Yes	Yes	113	122	No	No

18	no		040	yes	yes	98	108	no	no

### Establishment of cell lines

Fresh tumour tissue samples obtained during surgery were immersed immediately in buffered balanced saline. The tissues were washed several times, trimmed and minced into 1 to 2 mm pieces, which were placed in T25 tissue culture flasks with DMEM supplemented with 2 mM L-glutamine and 10% foetal bovine serum (FBS). The flasks were incubated at 37°C in an atmosphere containing 5% carbon dioxide. Primary tissue culture flasks were observed daily. To reduce the fibroblast growth, DMEM D-valine was added instead of L-valine. Fibroblasts were also removed mechanically and by brief exposure to trypsin (0.1%) and EDTA (0.02%). Tumour cells were subcultured when the flasks were 50% confluent. After three or four passages the cells were stored in liquid nitrogen. Low passage numbers (<30) were used for this study.

### Growth characteristics

The cell lines were denoted LU-HNSCC 3 to 8. All cell lines had an epitheloid appearance, and grew in a typical cobblestone pattern indicating squamous cell carcinoma origin. The cells differed in size and grew in colonies with cell-to-cell contact into confluence. All cell lines, besides number 6, grew as monolayer cultures and were easy to detach using trypsin; cells from LU-HNSCC 6 were more difficult to detach and to grew in multiple layers.

### Growth rate

To determine the *in vitro *tumour cell growth rate, 15,000–100,000 cells were plated in Petri dishes, and the number of cells was counted every second day in a Bürker chamber. The growth rate for each cell line was determined at least twice and the results were found to be reproducible. The mean values of 2–5 samples were estimated. The doubling times were derived from the exponential growth phase, and are given in Table 3, together with other data.

**Table 3:** Characteristics of the established cell lines regarding cisplatin sensitivity and cell doubling time.

**Cell line Name**	**Cisplatin IC50**	**Cell doubling time**
LU-HNxSCC	(μM) *	(Days) **

3	24,8 ± 6,4	1,8 ± 0,4

4	6 ± 0,9	1,1 ± 0,1

5	29,2 ± 3,1	1,6 ± 0,2

6	16,5 ± 4,5	1,3 ± 0,4

7	11,3 ± 3,5	2,2 ± 0,2

8	9,3 ± 3,1	1,4 ± 0,3

* cisplatin sensitivity is the mean of 3–6 experiments ± SEM and studied passage number 10–30

** cell doubling time is the mean values from two or more experiments and studied passage number 5–26

### Tumorigenicity in nude mice

To verify the malignancy of the established cell lines *in vitro*, a cellsolution containing the same cell amount from each cell line were injected subcutaneously into the lateral thoracic wall of nude mice. Tumour formation was observed for all cultured cell lines. The purpose of this experiment was to confirm the malignant characteristics of the cultured cell lines and to exclude a fibroblast cell population. The tumour formations in nude mice were no further examined in this experiment. The study was approved by the Regional Ethics Board of Southern Sweden Committe(LU376-01, M48-06).

### Flow cytometry

Frozen samples from 16 biopsies from primary tumours were analysed, and two samples from formalin-fixed and paraffin-embedded specimens were also analysed. Flow cytometry DNA analysis was performed as previously described [4]. Briefly, the tumour samples were minced, forced through a nylon net (pore size 140 μm, Tidbeck AB, Stockholm, Sweden), and fixed in 70% ethanol. The two formalin-fixed samples were processed to form cell suspensions according to a previously described method [5].

The separated cells were then treated with ribonuclease (Sigma-Aldrich, Stockholm, Sweden), incubated with pepsin (Merck, Darmstadt, Germany), and stained with propidium iodide (Sigma-Aldrich, Stockholm). Human lymphocytes were processed in parallel with the tumour samples and used as an external diploid control for the fresh samples. Flow cytometric DNA analysis was performed in a FACS Caliber (Becton, Dickinson, BD Biosciences, USA). Up to 20,000 nuclei were analysed from each sample. The DNA histograms obtained were automatically processed using Modfit LT 3.1™ software. The DNA index (DI) was calculated as the ratio of the modal channel values of the G0 and G1 peaks. By definition, the tumours manifesting a single DNA population were classified as diploid (i.e. DI = 1.00), and tumours manifesting two or more populations as non-diploid. The S-phase fraction (Spf) was estimated assuming that the S-phase compartment constituted a rectangular distribution between the modal values of the G0/G1 and G2 peaks.

### Chromosome banding analysis

Fresh samples from all but one of the 18 primary tumours previously had been subjected to short-term culturing and G-banding analysis [6]. All six established cell lines were also cytogenetically analysed using the same methods as in the present study.

### Immunohistochemistry

Immunohistochemical (IHC) analysis was performed on paraffin-embedded specimens to detect cyclin D1 (*CCND1*) expression. A commercial monoclonal antibody (NCL-cyclin D1, Novo) was used at a dilution of 1:20. A specimen known to be strongly positive, previously collected from a patient, was used as a positive control. The IHC results were scored as follows: A-negative; B 1–5% of the tumour cells positive; C 6–50% positive; D >50% positive. The negative controls were tested without primary antibodies.

### Fluorescence *in situ *hybridization

Fluorescence *in situ *hybridization (FISH) was performed as previously described [7], with minor modifications. Briefly, tumour cells were spread onto Superfrost Plus slides (Menzel, Braunschwieg, Germany), and then air dried and fixed in a series of 50, 75 and 100% Carnoy's solution (100% Carnoy's = 3:1 methanol:acetic acid). Prior to hybridization, the slides were denatured in 70% formamide, 2 × SSC, pH 7.0, at 72°C for three minutes, and dehydrated in a series of ethanol solutions (70, 85 and 100%). Two-colour FISH was performed with directly labelled probes for CCND1 and the centromere of chromosome 11 (LSI Cyclin D1 spectrum orange TM/CEP 11 spectrum green TM DNA Probe; Vysis, Inc., Downers Grove, IL, USA). Slides were counterstained with 0.2 mM 4,6-diamidino-2-phenylindole in an antifade solution (Vectashield, Vector H1000; Vector Laboratories, Burlingame, CA, USA) in order to visualize the nuclei and to prevent the fluorochromes from fading. A Zeiss Axioplan 2 microscope (Carl Zeiss AG, Oberkochen, Germany), equipped with a cooled CCD camera (Sensys; Photometrics, Tucson, NV, USA), operated by Quips FISH image analysis software (Vysis, Inc.) was used to analyse the samples. Hybridization signals from at least 50 nuclei were scored to assess the centromere and CCND1 copy numbers. The nuclei were defined as carrying an amplification if the number of gene probe signals divided by the number of centromere signals was ≥ 1.5.

### PCR-SSCP and DNA sequencing

Polymerase chain reaction-single-strand conformation polymorphism (PCR-SSCP) analysis and DNA sequencing were used to study the occurrence of mutations in exons 4–11 of *TP53*, as previously described [8]. Briefly, DNA was extracted using standard methods and used in a polymerase chain reaction to amplify the entire coding region of the *TP53 *gene in seven or eight different fragments. The PCR products were screened for mutations using SSCP. Samples showing altered mobility shift in SSCP were further analysed with direct DNA sequencing to determine the exact location and type of mutation.

### Cisplatin-induced cell death

The cell lines LU-HNSCC 3–8 were harvested by trypsinization, counted and seeded (10,000–26,000 cells/well) in 24-well plates, and allowed to grow for two days as monolayer cultures in DMEM medium (GIBCO, San Diego, CA, USA), supplemented with 10% FBS and antibiotics (100 U/ml streptomycin sulphate, GIBCO), under a 5% CO_2 _atmosphere at 37°C. On day two, cisplatin (Pharmalink AB, Upplands Väsby, Sweden) was added in serum-free medium, and the cells were incubated for 1 h at concentrations ranging from 0 to 100 μM. Thereafter, the drug-containing medium was removed, and cells were allowed to grow in drug-free medium for 5 days. On day 7, the cell viability was estimated by the crystal violet

assay, as described previously [9]. Briefly, the cells were incubated with 0.5% crystal violet (methanol:water,1:4) and excess dye was removed. The cells were solubilized by the addition of 0.10 M citrate buffer (SIGMA) (50% (v/v) ethanol) and then transferred to a new 96-well plate, and the absorbance was determined spectrophotometrically at 570 nm on a Multiscan MS (Labsystems, Finland) and corrected for background absorbance.

### 18F-FDG measurements

The established cell lines LU-HNSCC 3–8 were harvested by trypsinization, counted and seeded (50,000–250,000 cells/Petri dish) on day 0. The cells were allowed to grow for two days as monolayer cultures in DMEM medium(GIBCO, San Diego, CA) supplemented with 10% heat-inactivated FBS containing an antibiotic (GIBCO)(100 U/ml streptomycin sulphate), under a 5% CO_2 _atmosphere at 37°C. On day three, 2 ml 18F-FDG solution (0.62–1.33 MBq/ml) was added. After an hour the solution was removed by aspiration. The Petri dishes with cells were rinsed three times with PBS. The cells were then harvested from the Petri dishes by trypsinization and neutralized with 4 ml medium, and collected as samples for 18F-FDG determination together with the discarded 18F-FDG solution.

The 18F-FDG uptake in the cells and in the washing fractions was estimated using a calibrated 3 x 3 inch NaI(TI) well counter (in house) (1282 CompuGamma CS, LKB Wallac, Turku, Finland) and all 18F-FDG values were normalized for time. Electronic cell counting was performed using a NucleoCounter™ (Chemotec A/S, Allerod, Denmark) with the NucleoView™ software. The total cell content and number of viable cells were calculated per ml and correlated to the 18F-FDG uptake corrected for decay. This experiment was repeated in a second series.

### Statistics

Descriptive statistics, Fisher's exact test, and the chi-squared test for trend, were used to evaluate differences in clinical parameters between patients whose tumours could be cultured, and those that could not. Disease-free periods and overall survival time in these groups were examined using Kaplan-Meier graphs and log-rank tests (SPSS for Windows version 14.0, Chicago, IL, USA). The degree of linear relationship between pairs of variables measured on a continuous scale was summarized using correlation (r) and a test for zero slope in a corresponding linear regression model. Kruskal-Wallis' test was used to test the null hypothesis of equal cisplatin sensitivity for the cell lines. For comparison of 18F-FDG uptake between the cell lines, the following multiple linear regression model was used:

FDG = c_1 _+ b_1_V + c_2_I_2 _+ b_2_I_2_V + c_3_I_3 _+ b_3_I_3_V + c_4_I_4 _+ b_4_I_4_V + c_5_I_5 _+ b_5_I_5_V + c_6_I_6 _+ b_6_I_6_V

where the dependent variable 18F-FDG is 18F-FDG uptake and the independent variables are:

V = Number of viable cells

five dummy variables contrasting cell lines 2–6 to cell line 1:

I_j _= 1 if cell line = j, j = 2–6

I_j _= 0 otherwise

and five interaction parameters (products):

I_j_V = V if cell line = j, j = 2–6

I_j_V = 0 otherwise

This linear model has 12 parameters with the following interpretation:

c_1_: Intercept for the reference cell line (1)

b_1_: Slope for the reference cell line (1)

c_j_: Intercept difference between cell line j and the reference cell line, j = 2–6

b_j_: Slope difference between cell line j and the reference cell line, j = 2–6

In this modelling framework, an F-test was used to test the null hypothesis of equal 18F-FDG uptake for the cell lines at a fixed number of viable cells. The packages SPSS 14.0 (Chicago, IL, USA) and Stata 10.0 (StataCorp 2007, College Station, TX, USA) were used for statistical analysis.

## Results

### Patients: primary tumour characteristics and clinical course

Six new permanent squamous cell carcinoma lines *in vitro *were established from 18 HNSCCs, which constitutes an overall success rate of 33%.

**Figure 1:**
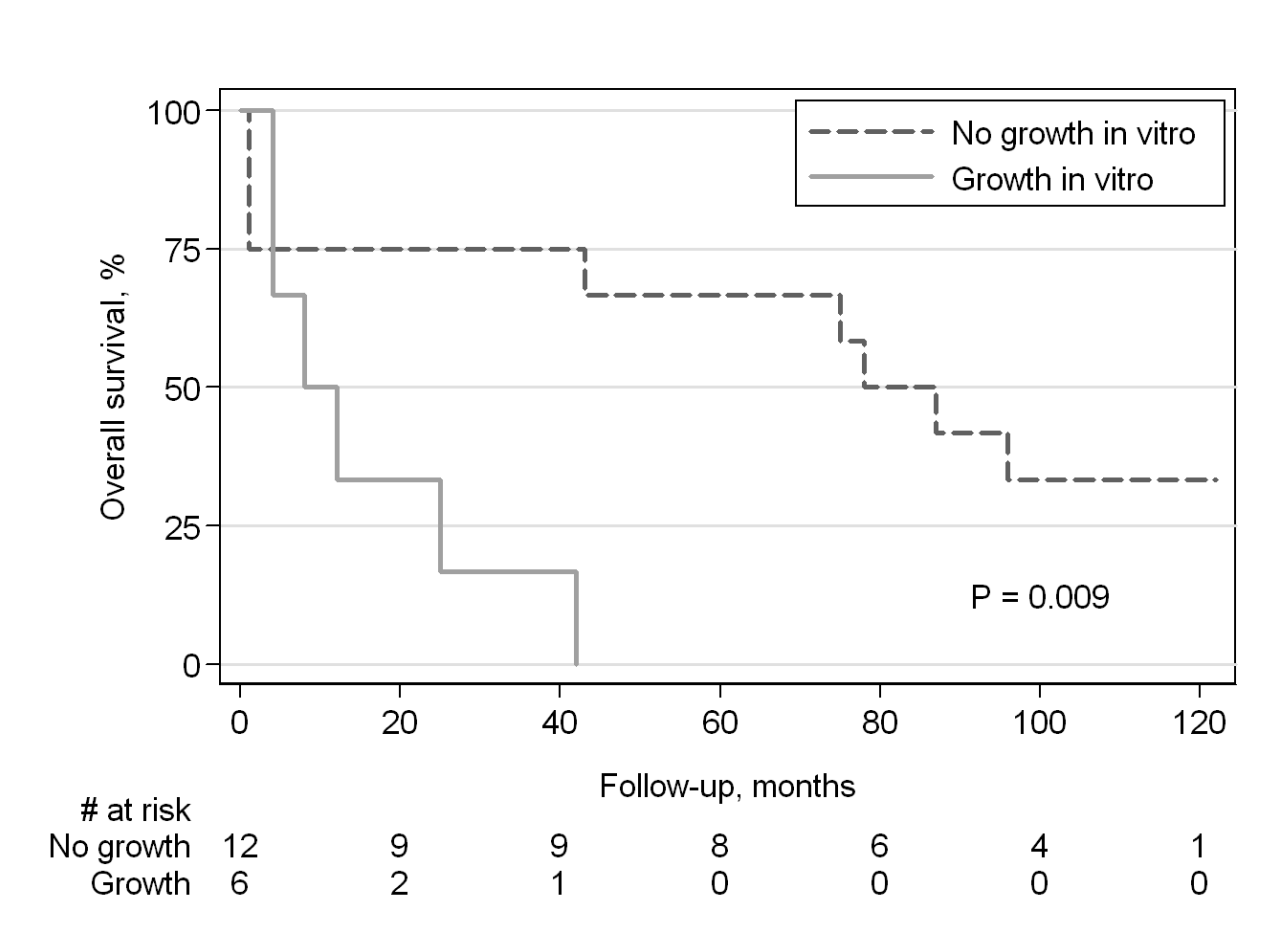
**Overall survival of the patients stratified by propensity of their tumours to grow *in vitro***. Survival times were calculated from date of diagnosis. Four patients were still alive (survival >100 months) when this analysis was carried out.

The overall survival of the patients as a function of the propensity of their tumours to grow *in vitro*, calculated from date of diagnosis, is shown in Figure 1. The outcome for the patients from whom cell lines could be established was worse than for the other patients; the median overall survival being 8 vs. 78 months (p = 0.009;logrank test), and the fraction of 5-year survival 0 vs. 67%. The mean disease-free survival time was 13 months for the patients whose tumours grew as cell lines, compared with 80 months for those whose cancers did not grow *in vitro *(p = 0.056). No differences were observed in the two groups regarding tumour site, TNM status, stage, grade, ploidity or DNA indices (data not shown).

### Chromosome banding analysis

Five of the six tumours that grew as cell lines had complex karyotypes, defined here as at least three different aberrations, when examined after short-term culturing. In contrast, only three of the 11 tumours from which no cell line could be established, and from which material had been sent for short-term culturing, had complex karyotypes. The remaining eight cases either failed to grow *in vitro *(seven cases), or showed an abnormal karyotype with simple changes (one case). There was no aberration in common among the tumours that yielded viable cell lines, and it was noted that none of them displayed homogeneous staining regions. Only minor changes were noted when comparing the karyotypes obtained after short-term culturing of primary tumours and in the corresponding cell lines (data not shown).

### The established cell lines

Three cell lines showed *TP53 *mutations, two in exon 7 and one in exon 5 (Table 4). Two of the mutations, one in exon 5 and one in exon 7, were missense mutations, and one in exon 7 was a deletion. Two of the three cell lines with *TP53 *mutation also showed *CCND1 *overexpression; one of these had *CCND1 *amplification according to FISH. No cell line showed *CCND1 *amplification without *TP53 *mutation.

**Table 4:** Characteristics of the established cell lines regarding proliferation parameters, DNA content and gene expression.

**Cell line**	**Flowcytometry** (n = 1)			**Immunohistochemistry** (n = 3)	**PCR_SSCP** (n = 1)	**FISH** (n = 1)	**Cytogenetics** (n = 1)
**LU-HNxSCC**	**Ploidity**	**DNA indices**	**S%phase**	**Cyclin D1**	**Tp53**	**Cyclin D1**	

3	Diploid (p4)	1	ND*	A	0	0	not complex

4	Nondilploid (p22)	1,4	26,3	C	exon7 R249G	0	complex

5	Nondiploid (p27)	2,1	23,8	D	exon5 H168P	++	complex

6	Nondiploid (p20)	1,2	16	A	0	0	complex

7	Nondiploid (p6)	1,4	9,8	A	0	deletion	complex

8	Nondiploid (p22)	1,6	22,2	B(1/3C)	exon7 Ldel252	1/3+	complex

* ND = not determined

### 18F-FDG uptake

**Figure 2:**
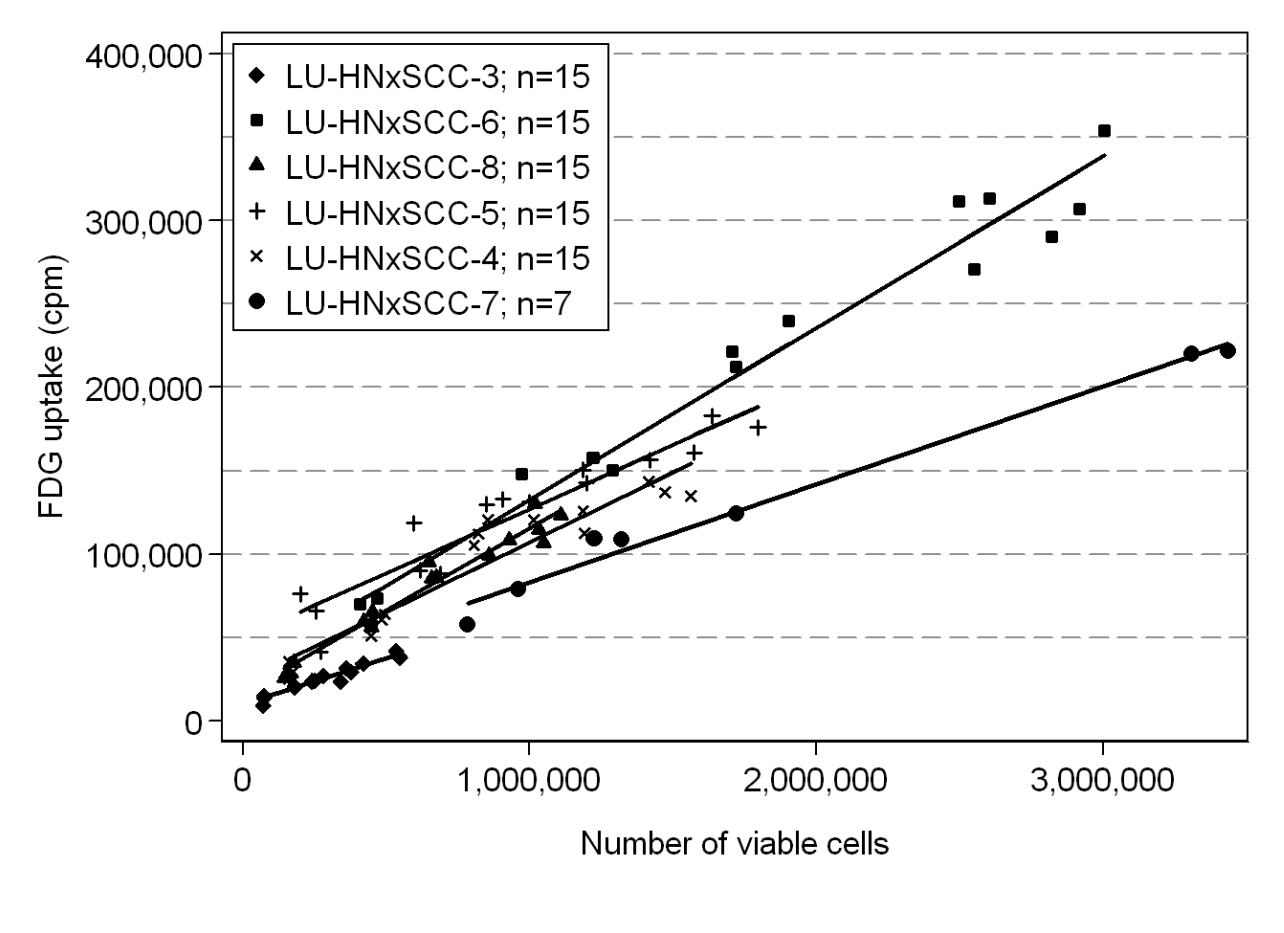
**The 18F-FDG uptake, expressed as counts per minute adjusted for time, versus number of viable cells present for the six cell lines, scatter plot and fitted regression lines**.

The 18F-FDG uptake, expressed as counts per minute (cpm) adjusted for time, was strongly correlated with the number of viable cells present, as illustrated in Figure 2. The correlations varied between 0.94 (LU-HNSCC 3) and 0.99 (LU-HNSCC 7). The null hypothesis of no difference in 18F-FDG uptake between the cell lines was evaluated in a linear regression framework (see Statistics) and according to this model, the predicted 18F-FDG uptake for 1,000,000 viable cells varied more than a factor 2, from 65,000 cpm for LU-HNSCC 3 to 133,000 cpm for LU-HNSCC 6. The null hypothesis of equal 18F-FDG uptake for this fixed number of viable cells could be rejected (p < 0.0001; F-test). Significant differences in 18F-FDG uptake between the cell lines (p < 0.01) was seen for all reference values from 50,000 to 1,500,000 viable cells and also in sub-group analyses excluding one of the two cell lines with non-overlapping ranges for number of viable cells.

No correlation was observed between 18F-FDG uptake or gene expression and cell characteristics, such as *TP53 *and *CCND1 *status or DNA ploidity.

We found an inverse correlation (r = -0.82) between cell doubling time (DT) and 18F-FDG uptake; the shorter the doubling time, the higher the 18F-FDG uptake (p = 0.04; test for zero slope in a linear regression of predicted 18F-FDG uptake at 1,000,000 viable cells on doubling time; n = 6).

This inverse relationship was even stronger if the cell line LU-HNSCC 3 with no observations above 600,000 viable cells was omitted (r = -0.95; p = 0.01) or if the cell line LU-HNSCC 7 with no observations below 700,000 viable cells was omitted and the 18F-FDG uptake was predicted for 500,000 viable cells (r = -0.96; p = 0.01). The experiment was repeated with similar results.

In brief, the correlations between 18F-FDG uptake and number of viable cells varied from 0.81 to 0.98 and the predicted 18F-FDG uptake at 1,000,000 viable cells varied significantly between the cell lines also in the second experiment (p < 0.0001). Also the negative correlation between 18F-FDG uptake and DT was reproduced in the second series (r = -0.70; p = 0.12; n = 6). By combining the data from the two experiments, the p-value for the inverse correlation between 18F-FDG uptake and DT dropped to 0.004.

### Cisplatin sensitivity

**Figure 3:**
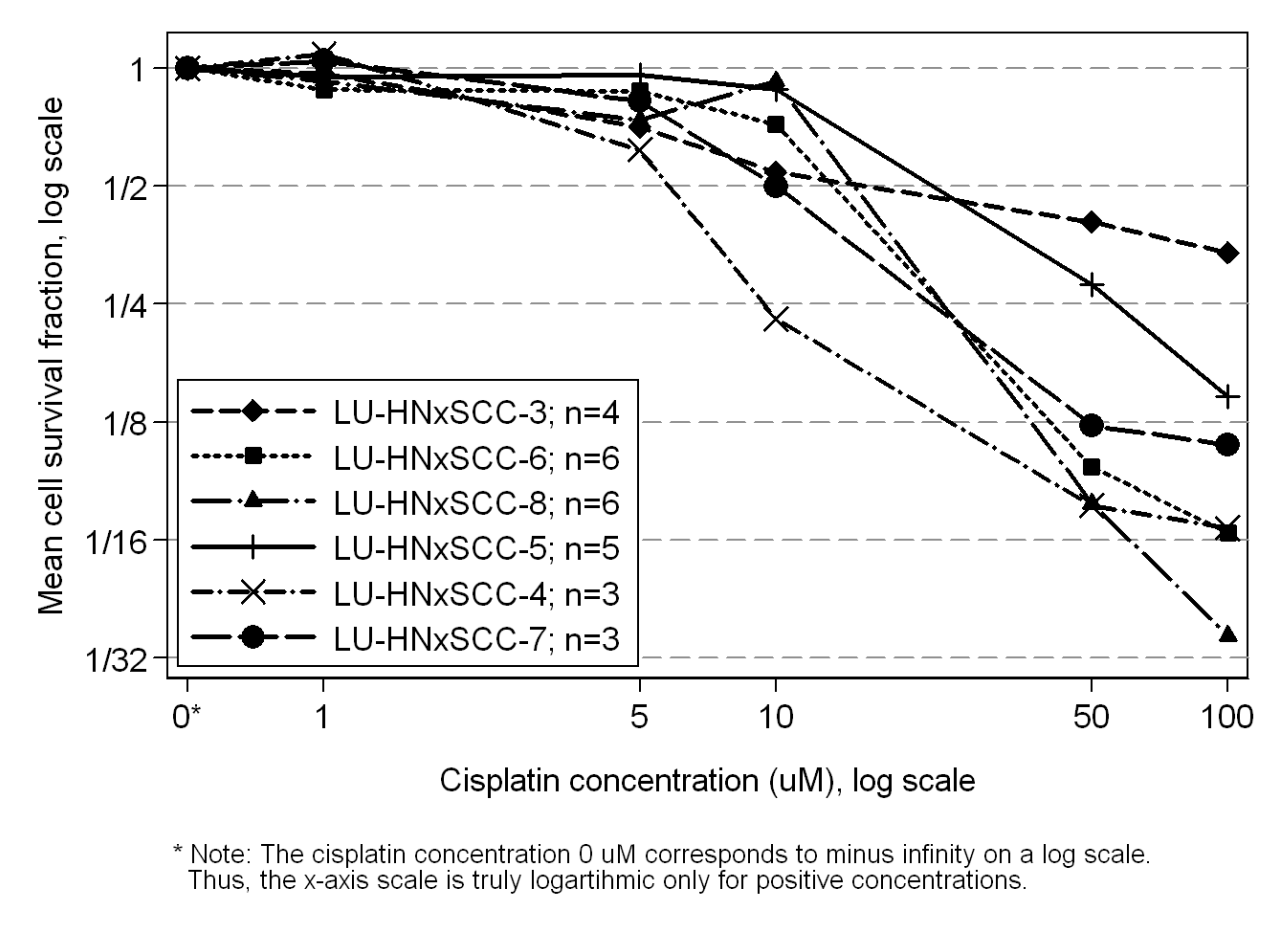
**Survival curves of the different cell lines exposed to varying concentrations of cisplatin obtained by crystal violet assay**. Each value represents an average of at least three experiment.

The cisplatin sensitivity of the different cell lines is illustrated in Figure 3. Significant differences in cisplatin sensitivity between the cell lines was seen at 5, 50 and 100 μM (p < 0.0001; Kruskal-Wallis test). The values of IC_50 _for the different cell lines varied between 6 and 29 μM. The cisplatin sensitivity did not show any relationship with *TP53 *mutations, *CCND1 *amplification or overexpression, or tumour doubling time.

## Discussion

In accordance with other studies [10-12], we found that tumours that could grow *in vitro *were more aggressive in their biological behaviour, with shorter patient disease-free periods and overall survival time, compared with those that did not grow *in vitro*. No correlation was found between ability to grow and clinical parameters such as TNM status, or tumour grade or site. In agreement with our results, Kim et al. established nine new permanent SCC cell lines, but their propensity to grow *in vitro *did not appear to be related to tumour site or grade [13]. Thus, *in vitro *growth, in the present study seems to be an independent prognostic factor, in concordance with other authors [10-12] although there also are reports on lack of such correlation [14]. The capacity of tumour cells to grow *in vitro *could be dependent on their genetic alterations. Support for this hypothesis comes from the finding that all the culturable cell lines, except for one in this study were seen to have complex karyotypes after short-term culturing. Additionally, complex karyotypes have been shown to be associated with poor prognosis [15].

We also investigated possible differences between the two tumour groups regarding DNA content, index and S-phase fraction, but no statistically significant differences were found. These cellular characteristics have been widely investigated previously, since they are assumed to reflect the loss of normal cell proliferation control and the underlying genetic abnormalities. The prognostic value of DNA content is, however, more uncertain. While some studies have found a correlation with poor outcome and higher recurrence rate in aneuploid tumours [16,17], the opposite, i.e. better survival of those with non-diploid tumours, has also been reported [18].

The extent of 18F-FDG uptake has been suggested to provide a measure of tumour aggressiveness, and thus to be associated with poor prognosis in many tumour types [19,20], including HNSCC [21,22]. The usefulness of 18F-FDG-PET in HNSCC for detection of recurrent disease is well recognized and clinical studies have shown a capacity for PET to predict response to cytotoxic therapy [23,24]. We determined the 18F-FDG uptake and its relation to cell viability in the established cell lines and found an inverse correlation between cell doubling time (DT) and 18F-FDG uptake; the shorter the doubling time, the higher the 18F-FDG uptake. The correlation between the number of viable cells and 18F-FDG uptake, and between a shorter tumour doubling time and a higher 18F-FDG uptake, support a relation between 18F-FDG metabolism and tumour aggressiveness. A similar correlation between 18F-FDG uptake and cell proliferation has been described for other cancer types, including breast and colonic tumours [25]. In another *in vitro *study using HNSCC lines, Minn et al.[26] found a relation between 18F-FDG uptake and cell proliferation index, defined as the percentage of tumour cells in the S+G2/M phase, while Smith et al.[27] found a similar correlation with the S-phase fraction. Furthermore, in a clinical trial on 14 patients, a close correlation between growth fraction, determined by Ki67-MIB-1, and PCNA, assessed with immunohistochemistry, and 18F-FDG uptake was demonstrated [28], but no correlation between 18F-FDG uptake and DNA ploidity was seen. The close relation between *CCND1 *status and cell proliferation suggests that deregulated *CCND1 *could be a factor affecting 18F-FDG uptake. However, we found no correlation between cyclin D1 expression or *CCND1 *amplification and 18F-FDG uptake. Similar results, i.e. no correlation between *CCND1 s*tatus and 18F-FDG uptake, have been reported in a clinical trial on lung cancer patients [29].

Some studies have found *TP53 *mutations to be accompanied by increased glycolysis, which could be the result of reduced synthesis of proteins in the COX ∏ subunit or increased transcription of HK-2 [30,31]. We found no association between the presence or absence of *TP53 *and increased 18F-FDG uptake.

To best of our knowledge, there are no previous reports on possible correlations between 18F-FDG uptake and *CCND1 *or *TP53 *status in HNSCC, although similar studies have been carried out on other tumours [29,30], demonstrating a correlation between *TP53 *overexpression and higher 18F-FDG uptake [30]. One reason why we did not observe any correlation between the 18F-FDG uptake and the *TP53 *and *CCND1 *status could be that the tumour cells *in vitro *have an excess of nutrients, and that they must be placed under stress to reveal a correlation. Therefore, the next experimental step will be to treat the cell lines with cisplatin, perhaps providing more insight into the complex and still enigmatic mechanisms behind the intracellular uptake and accumulation of 18F-FDG.

The six cell lines were also tested regarding cisplatin sensitivity. Cisplatin-induced cell death was measured using crystal violet staining, a method evaluated before [9]. A statistical difference was found between the cell lines, demonstrating the usefulness of the model for studying chemosensitivity.

## Conclusion

The results in this present study support the value of tumour cell cultures as a model for prognostic and predictive studies. We found the successful establishment of an *in vitro *cell line from a tumour to be an independent negative prognostic marker. Furthermore, we found it feasible to study metabolic activity with 18F-FDG uptake, and other tumour biologic characteristics, including the chemosensitivity of the cell lines. Despite the relatively small number of tumour lines, we found a statistically significant correlation between a shorter tumour doubling time and higher 18F-FDG uptake. However, no significant difference was seen between 18F-FDG uptake and other proliferation parameters, including *TP53 *and *CCND1 *status. Although, the complex metabolic interactions between host and tumour, which create the microenvironment *in vivo*, will not be reproducible in cultured cell lines the growing knowledge of tumour cell characteristics will provide more understanding of the clinical behaviour of HNSCC tumours and of prognosis and therapy results for HNSCC patients.

## Competing interests

The authors declare that they have no competing interests.

## Authors' contributions

EH participated in the experiments in vitro, interpretation of the study and drafted the manuscript. EK conceived of the study, and participated in its design and interpretation. BB performed the flowcytometry analysis and the interpretation. PB performed the statistically analyses and interpretation. AB analysed the PCR-SSCP and DNA sequencing and interpretation. EB participated in the design of the study and revising the manuscript. FM evaluated and analysed the cytogenetic results. TO performed the FDG uptake measurements and interpretation. KR performed the FISH method and evaluation. JW participated in its design and coordination. PW conceived of the study, participated in its design and coordination and helped to draft the manuscript. All authors read and approved the final manuscript.
